# One-Carbon Metabolism Inhibition Depletes Purines and Results in Profound and Prolonged Ewing Sarcoma Growth Suppression

**DOI:** 10.1158/2767-9764.CRC-25-0218

**Published:** 2025-08-08

**Authors:** Sara Zirpoli, Noah Copperman, Shrey Patel, Alexander Forrest, Zhanjun Hou, Larry H. Matherly, David M. Loeb, Antonio Di Cristofano

**Affiliations:** 1 Department of Developmental and Molecular Biology, Albert Einstein College of Medicine, Bronx, New York.; 2 Department of Molecular Medicine and Medical Biotechnology, University of Naples Federico II, Naples, Italy.; 3 Department of Pediatrics, Albert Einstein College of Medicine, Bronx, New York.; 4 Department of Oncology, Wayne State University School of Medicine, and the Barbara Ann Karmanos Cancer Institute, Detroit, Michigan.; 5 Cancer Dormancy and Tumor Microenvironment Institute, Albert Einstein College of Medicine, Bronx, New York.; 6 Montefiore Einstein Comprehensive Cancer Center, Albert Einstein College of Medicine, Bronx, New York.; 7 Marilyn and Stanley M. Katz Institute for Immunotherapy for Cancer and Inflammatory Disorders, Albert Einstein College of Medicine, Bronx, New York.

## Abstract

Ewing sarcoma is the second most common primary bone malignancy in adolescents and young adults. Patients who present with localized disease have experienced a steadily improving survival rate over the years, whereas those who present with metastatic disease have the same dismal prognosis as 30 years ago, with long-term survival rates of less than 20%, despite maximal intensification of chemotherapy. Thus, novel treatment approaches are a significant unmet clinical need. Targeting metabolic differences between Ewing sarcoma and normal cells offers a promising approach to improve outcomes for these patients. One-carbon metabolism utilizes serine and folate to generate glycine and tetrahydrofolate-bound one-carbon units required for *de novo* nucleotide biosynthesis. Elevated expression of several one-carbon metabolism genes is significantly associated with reduced survival in patients with Ewing sarcoma. We show that both genetic inhibition and pharmacologic inhibition of a key enzyme of the mitochondrial arm of the one-carbon metabolic pathway, serine hydroxymethyltransferase 2, lead to substantial inhibition of Ewing sarcoma cell proliferation and colony-forming ability and that this effect is primarily caused by depletion of glycine and one-carbon units required for the synthesis of purine nucleotides. Inhibition of one-carbon metabolism at a different node, using the clinically relevant dihydrofolate reductase inhibitor pralatrexate, similarly yields profound growth inhibition, with depletion of thymidylate and purine nucleotides. Genetic depletion of serine hydroxymethyltransferase 2 dramatically impairs tumor growth in a xenograft model of Ewing sarcoma. Together, these data establish dependence on one-carbon metabolism as a novel and targetable vulnerability of Ewing sarcoma cells, which can be exploited for therapy.

**Significance:**

Using both genetic and pharmacologic approaches, this study identifies Ewing sarcoma’s dependence on one-carbon metabolism as a targetable vulnerability that can be effectively harnessed for therapy.

## Introduction

Ewing sarcoma is the second most common bone tumor in children, adolescents, and young adults. Patients who present with localized disease have experienced steady improvements in survival as a series of clinical trials have demonstrated the efficacy of increased chemotherapy intensity (1). In contrast, patients who either present with metastatic disease or who suffer a relapse have the same grim prognosis as in the 1980s, with no improvement in survival despite numerous clinical trials aimed at maximizing chemotherapy intensity (2). For these patients, it is clear that future improvements in patient outcomes will not derive from modifying their chemotherapy but rather from identifying alternative therapeutic approaches based on a deeper understanding of Ewing sarcoma biology. One promising approach is to identify metabolic differences between normal cells and Ewing sarcoma cells that can be exploited for therapeutic gain.

**Figure 1:**
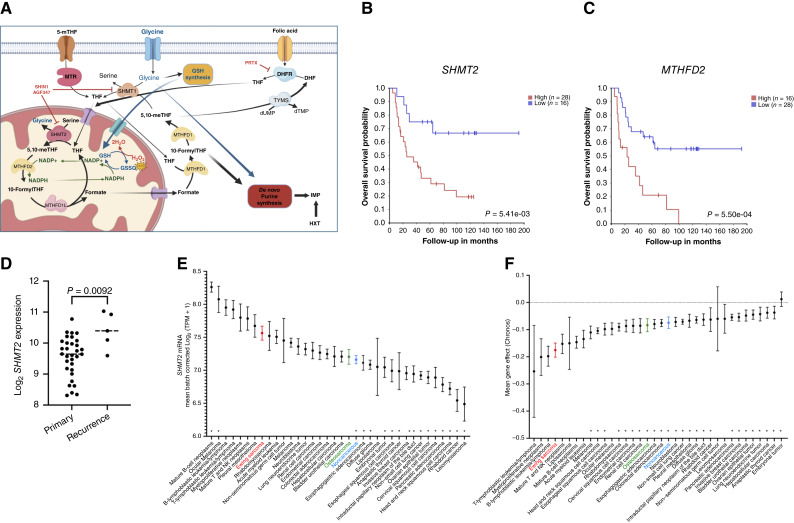
High expression of *SHMT2* and *MTHFD2* is associated with more aggressive clinical features. **A,** Cartoon depicting the key steps of the cytoplasmic and mitochondrial arms of one-carbon metabolism, as well as the targets of the three inhibitors utilized in this study. 5,10-meTHF, 5,10-methylene-tetrahydrofolate; 5-mTHF, 5-methyl-tetrahydrofolate; DHF, dihydrofolate; GSH, glutathione; GSSG, glutathione disulfide; HXT, hypoxanthine; IMP, inosine monophosphate; MTR, methionine synthase; NADP, nicotinamide adenine dinucleotide phosphate; TYMS, thymidylate synthase. **B** and **C,** Overall survival of patients with Ewing sarcoma (*n* = 44) expressing high or low levels of *SHMT2* (**B**) or *MTHFD2* (**C**). **D,** Expression levels of *SHMT2* in patients with primary (*n* = 32) and recurrent (*n* = 5) disease. Analysis was performed on dataset GSE17679 using R2: Genomics Analysis and Visualization Platform (http://r2.amc.nl). **E,** Expression of *SHMT2* in cell lines from different tumor types. Analysis was performed using Data Explorer 2.0 and the DepMap Public 25Q2 Dataset (depmap.org), selecting only tumor types with at least 10 cell lines. *, *P* < 0.05 vs. Ewing sarcoma. **F,** Analysis of the effect of CRISPR/Cas9-mediated SHMT2 deletion on the viability of cell lines from different tumor types. 0, no effect; −1, an essential gene. Analysis was performed as in **E** using the DepMap Public 25Q2 Chronos Dataset. *, *P* < 0.05 vs. Ewing sarcoma.

The primary oncogenic mutation driving Ewing sarcoma is a chromosomal translocation, most frequently t(11;22), resulting in the production of the Ewing sarcoma::FLI1 oncoprotein that has both transcriptional regulatory and mRNA splicing activity (3). The literature suggests that the Ewing sarcoma–FLI1 fusion oncogene directly regulates the expression of several enzymes involved in *de novo* serine and glycine biosynthesis and in one-carbon metabolism (4, 5). Furthermore, inhibition of serine synthesis at phosphoglycerate dehydrogenase in Ewing sarcoma cells significantly inhibits cell proliferation in the absence of exogenous serine and glycine, in line with a critical role of one-carbon metabolism in supporting Ewing sarcoma cell growth (6). The one-carbon metabolic pathway utilizes serine and dietary folate to generate glycine and tetrahydrofolate (THF)-bound one-carbon units, which are essential for a variety of cellular processes, including *de novo* nucleotide biosynthesis, NADPH and glutathione production, methionine synthesis, biological methylation, and mitochondrial protein translation (7–9). One-carbon metabolism has two compartmentalized arms: one oxidative in the mitochondria, driven by serine hydroxymethyltransferase 2 (SHMT2), 5,10-methylene THF dehydrogenase 2 (MTHFD2), and MTHFD1-like (MTHFD1L), and one reductive in the cytoplasm, driven by SHMT1 and MTHFD1. These two arms form a normally unidirectional cycle between the two cellular compartments (Fig.1A; ref. 10).

*SHMT2* and *MTHFD2* are among the most consistently upregulated metabolic genes in cancer (11, 12), supporting the critical role of one-carbon metabolism in the production of metabolites to fuel rapid growth and cell division and to survive intra- and extracellular oxidative stress. The present study combines genetic and pharmacologic approaches to explore the role of one-carbon metabolism in Ewing sarcoma and demonstrates that Ewing sarcoma cells have an absolute dependence on mitochondrial one-carbon metabolism, which is essential for *de novo* nucleotide biosynthesis and glycine production. Furthermore, we provide compelling *in vivo* evidence that targeting one-carbon metabolism effectively impairs tumor growth in a xenograft model of Ewing sarcoma.

## Materials and Methods

### Cell culture

The human Ewing sarcoma cell lines used in this study (Supplementary Table S1) were maintained at 37°C with 5% CO_2_ in RPMI 1640 (Cytiva) with 10% FBS (Biowest). For all the experiments, cells were grown in RPMI 1640 with 10% dialyzed FBS (dFBS; Biowest). Cell lines were initially obtained from the Children’s Oncology Group biorepository in 2016. Their identity was validated by short tandem repeat profiling, repeated every 6 months. Cell lines are screened regularly for mycoplasma contamination and grown in the presence of MycoZap Plus-CL (Lonza).

### Short hairpin RNA–mediated gene knockdown

Cells were transduced with lentiviruses encoding short-hairpin RNAs (shRNA) against *SHMT1* (RHS4430-101028254, RHS4430-101032591, and RHS3979-9602175) and *SHMT2* (RHS3979-9602214 and RHS3979-9602215).

shRNAs for *SHMT2* were cloned into the Tet-pLKO-puro (Addgene, #21915; RRID:Addgene_21915). Lentiviral constructs were packaged in HEK293 cells (RRID:CVCL_0045) transfected using Lipofectamine 2000 (Invitrogen). Viral supernatants were collected 48 to 72 hours after transfection, combined, and filtered using 0.45 μm Nalgene SFCA syringe filters (Thermo Fisher Scientific). Target cells were plated 24 hours before infection and then exposed to viral supernatants supplemented with 8 μg/mL polybrene (Santa Cruz Biotechnology). Infected cell lines were then selected for at least 72 hours with 2 μg/mL puromycin (Corning).

Transduced cell lines were pretreated with 0.25 µg/mL doxycycline hyclate (Sigma-Aldrich) for 48 hours prior to plating for proliferation experiments. In all experiments performed on transduced cell lines, doxycycline hyclate was replaced every 48 hours.

### Drugs and chemicals

The following chemical compounds were used: SHIN1 (AOBIOUS), AGF347 (Sigma-Aldrich), hypoxanthine (TCI America), adenine, thymidine and glycine (Sigma-Aldrich), sodium formate (Thermo Fisher Scientific), and ethyl ester glutathione and pralatrexate (PRTX; Cayman Chemical).

### Incucyte proliferation assays

A total of 2,500 cells per well were plated in a 96-well plate (Corning) in 75 µL of RPMI 1640/10% dFBS and doxycycline and maintained under standard conditions. Plates were inserted into an Incucyte Live-Cell Analysis System, and confluence was measured utilizing live-cell time-lapse imaging.

### Viability assays

Cells were plated in clear bottom, black wall, 96-well plates in medium with dFBS (Biowest). Treatments were added 24 hours after plating in sextuplicate. alamarBlue was directly added to the culture medium of treated and control cells after 72 hours of treatment. Fluorescence was measured using a plate reader (excitation 530 nm, emission 590 nm). Statistical analysis and calculation of EC_50_ values were performed using GraphPad Prism (RRID:SCR_002798).

### Cell proliferation assays

Cells were plated in 12-well plates (5,000 cells) or 6-well plates (10,000 cells) in RPMI 1640 medium with dFBS. Treatments were added in triplicate 24 hours after plating. At the end of the experiment, cells were trypsinized and counted using a Coulter particle counter (Beckman Coulter).

### Colony formation assay

A total of 1,000 cells were plated in each well of a 12-well plate. Treatments were added in triplicate 24 hours after plating. Medium was replaced every 72 hours. Colonies were fixed with 10% neutral buffered formalin and stained with 0.01% crystal violet (Sigma-Aldrich). For quantitation, crystal violet was solubilized in 30% acetic acid, and absorbance at 560 nm was determined using a plate reader.

### Lactate dehydrogenase release assay

Cells were treated as described above (viability assay). After 72 hours, lactate dehydrogenase release was assessed using a commercial kit (LDH-Cytox Assay Kit, BioLegend), according to the manufacturer’s instructions.

### Cell-cycle analysis

For cell-cycle analysis, cells were treated with DMSO or inhibitors (SHIN1, 5 µmol/L; AGF347, 2 µmol/L; PRTX, 1 nmol/L), harvested after 48 hours by trypsin treatment, and fixed in 75% ethanol on ice for 4 hours. After washing with PBS, cells were stained with FxCycle PI/RNase staining solution (Invitrogen), and DNA content was measured using an Aurora system (Cytek Biosciences).

### Three-dimensional spheroid assay

A total of 200 cells were plated in 96-well round-bottom ultralow attachment plates (Corning) in RPMI 1640 with dFBS and centrifuged at 350 rpm for 5 minutes. Treatments were added 3 days after plating. Spheroids were microphotographed before treatment and 96 hours after treatment, and volume was measured using ImageJ (RRID:SCR_003070).

### Western blot

Cells were homogenized on ice in RIPA buffer supplemented with Halt Protease and Phosphatase Inhibitor Cocktail (Thermo Fisher Scientific). Protein concentrations were determined using the Pierce BCA Protein Kit (Thermo Fisher Scientific). Western blot analysis was conducted using 50 µg of protein on ExpressPlus precast gels (GenScript). Proteins were blotted onto polyvinylidene difluoride membranes (Millipore). The membranes were probed with the following antibodies: SHMT1 (Cell Signaling Technology, Cat. # 26080, RRID:AB_3698387), SHMT2 (Cell Signaling Technology, Cat. # 33443, RRID:AB_3683566), and actin (Santa Cruz Biotechnology, Cat. # sc-47778, RRID:AB_62663). All the primary antibodies were used at the vendor-suggested dilution in 5% BSA in tris-buffered saline with Tween 20. Signals were detected with horseradish peroxidase–conjugated secondary antibodies (Thermo Fisher Scientific) and the chemiluminescence substrate Luminata Crescendo (EMD Millipore).

### Metabolomics

SK-ES-1 cells (1 million cells/60 mm dish) were seeded in four 60 mm dishes in 5 mL of complete RPMI 1640 (10% dFBS). The cells were allowed to adhere for 24 hours. The cells were treated with 10 µmol/L SHIN1, 10 nmol/L PRTX, or a comparable volume of vehicle (1% DMSO) in the presence of adenosine (60 µmol/L) and thymidine (10 µmol/L). After 16 hours, the cells were washed with PBS (3×); the media were replaced with complete folate- and serine-free RPMI 1640 (containing 130 µmol/L glycine and 2.3 µmol/L folic acid) supplemented with 10% dFBS and [2,3,3-^2^H]serine (250 µmol/L), including drug or vehicle, as appropriate, in the presence of adenosine (60 µmol/L) and thymidine (10 µmol/L). The cells were incubated for 24 hours. The media were aspirated, and cells were washed (3×) rapidly (<30 seconds) with 5 mL ice-cold PBS; metabolism was quickly quenched with cold 800 µL methanol/water (80:20). The dishes were placed on a shaker and allowed to rock on dry ice for 10 minutes to cover the entire dish with cold 80:20 methanol/water to extract the metabolites and then harvested by scraping and pipetting the contents into 1.5 mL Eppendorf tubes. The tubes were centrifuged (4°C, 14,000 RPM, 10 minutes). The supernatants were collected and analyzed by reverse-phase ion-pairing chromatography coupled with negative-mode electrospray ionization high-resolution mass spectrometry on a stand-alone Orbitrap (Thermo Fisher Exactive). Raw metabolite values were adjusted to correct for normal ion distributions and normalized to total proteins from the postextraction pellet by solubilizing with 500 µL 0.5 N NaOH and using 20 µL for protein assay with the Folin phenol method for protein quantification.

### *In vivo* studies

Six- to eight-week-old NOD-*scid IL2r*^*null*^ mice (JAX #005557; RRID:IMSR_JAX:005557; five males and five females) were injected subcutaneously with 3 × 10^6^ SK-ES-vector or SK-ES-ish*SHMT2* cells resuspended in PBS. Doxycycline hyclate was dissolved in drinking water (2 mg/mL) and replaced every 3 days. Treatment started the day after injection. Tumor volume was calculated from two-dimensional measurements with an electronic caliper using the following formula: tumor volume = (length × width^2^) × 0.5, every 3 days. Tumor weights were measured at the end of the experiment. Data were plotted and analyzed using GraphPad Prism. All the animal studies were approved by the Einstein Institutional Animal Care and Use Committee.

### Statistical analyses

All data reflect at least three biological replicates, each with three to six technical replicates. Statistical analyses were done in GraphPad Prism using an unpaired Student *t* test or two-way ANOVA as appropriate.

### Data availability

The data generated in this study are available from the corresponding authors upon request.

## Results

### High expression of one-carbon metabolism genes is associated with poor survival in Ewing sarcoma

Analysis of an Ewing sarcoma gene expression dataset (GSE17679; ref. 13) annotated with clinical and survival data revealed that patients with tumors expressing high levels of RNAs for *SHMT2* or *MTHFD2*, which encode two key enzymes involved in the mitochondrial arm of the one-carbon metabolic pathway (Fig. 1A), experience dramatically reduced overall survival, whereas no such association was found for *SHMT1*. A trend toward reduced overall survival was also observed for patients with elevated expression of *MTHFD1* and *MTHFD1L* (Fig. 1A–C; Supplementary Fig. S1). Notably, *SHMT2* is also expressed at significantly higher levels in recurrent lesions compared with primary tumors (Fig. 1D). These data strongly suggest that increased expression levels and, consequently, activity of several enzymes of one-carbon metabolism are associated with Ewing sarcoma tumor growth and aggressiveness.

As the cell of origin of Ewing sarcoma is still a matter of debate, with recent data pointing to a mesenchymal or neural crest stem cell (bioRxiv 2024.10.27.620438; ref. 14), it is not possible to test whether one-carbon metabolism enzymes are overexpressed in Ewing sarcoma compared with its normal counterpart; however, when we analyzed the DepMap RNA sequencing dataset, containing data from more than 1,000 cell lines representative of a large number of different tumor types (15), we found that Ewing sarcoma cell lines display the third highest *SHMT2* expression among all solid tumors (Fig. 1E) and a much higher mean expression than nontransformed cell lines. Furthermore, when we analyzed the effect of CRISPR/Cas9-mediated gene inactivation on the viability of the 769 cell lines included in the DepMap Chronos dataset, we found that Ewing sarcoma ranks first among all solid tumors for dependence on SHMT2 (Fig. 1F). Notably, Ewing sarcoma has a higher dependence on SHMT2 than osteosarcoma, a tumor for which antifolates represent the first-line therapy (16).

### Genetic downregulation of *SHMT2* impairs the proliferation of Ewing sarcoma cells

**Figure 2:**
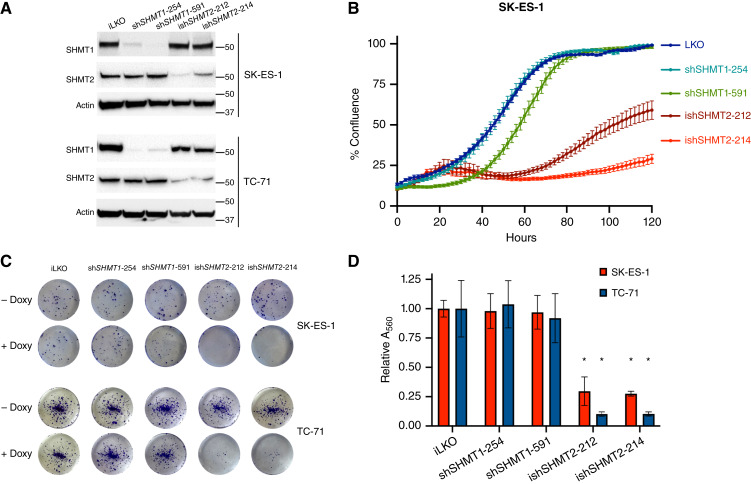
Depletion of *SHMT2*, but not *SHMT1*, impairs proliferation of Ewing sarcoma cells. **A,** shRNA-mediated depletion of *SHMT1* (constitutive, shSHMT1) or *SHMT2* (inducible, ishSHMT2) in SK-ES-1 and TC-71 cells. **B,** Incucyte live analysis of cell proliferation upon depletion of *SHMT1* and *SHMT2* with two shRNAs each. LKO: empty vector. **C,** Representative colony-forming assays of SK-ES-1 and TC-71 cells upon depletion of SHMT1 or SHMT2. **D,** Quantitative results from the experiment in **C**. *, *P* < 0.01.

To define the biological consequences of the overexpression of one-carbon metabolism genes, we generated two Ewing sarcoma cell lines (SK-ES-1 and TC-71) expressing control shRNAs or shRNAs targeting *SHMT1* or *SHMT2*. Notably, although we could readily generate cell lines constitutively expressing sh*SHMT1*, Ewing sarcoma cells did not survive constitutive *SHMT2* targeting such that we had to use a doxycycline-inducible vector for expressing these shRNAs (Fig. 2A). shRNA-mediated depletion of SHMT1 did not alter the proliferation of Ewing sarcoma cells. Knockdown of SHMT2 expression, however, significantly impaired cell proliferation (Fig. 2B; Supplementary Fig. S2).

To assess the long-term impact of genetic inhibition of the one-carbon pathway, we performed colony-forming assays using the SK-ES-1 and TC-71 cell lines. In line with the results of the short-term cell proliferation assays, shRNA-mediated depletion of SHMT1 did not alter the ability of Ewing sarcoma cells to form colonies. Conversely, depletion of SHMT2 dramatically inhibited colony formation (80%–90%, Fig. 2C and D). The inability to survive in the prolonged absence of SHMT2 suggests that Ewing sarcoma cells are “addicted” to elevated activity of SHMT2.

### Pharmacologic inhibition of one-carbon metabolism impairs the proliferation of Ewing sarcoma cells

**Figure 3:**
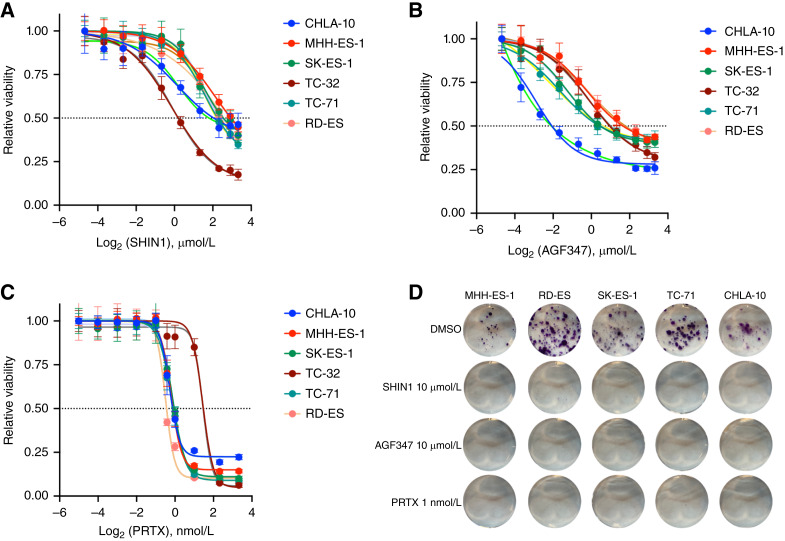
Pharmacologic inhibition of one-carbon metabolism impairs Ewing sarcoma cell proliferation. **A–C,** Viability, measured using alamarBlue, of six Ewing sarcoma cell lines treated for 72 hours with increasing concentrations of SHIN1 (**A**), AGF347 (**B**), and PRTX (**C**). **D,** Colony-forming assay using five Ewing sarcoma cell lines treated for 2 weeks with the indicated inhibitors.

In addition to the classical antifolates, methotrexate and pemetrexed, which primarily target dihydrofolate reductase (DHFR) and thymidylate synthase, respectively (17, 18), a number of novel selective inhibitors of enzymes involved in one-carbon metabolism have been developed in recent years (19). Of particular relevance to this study, SHIN1 was synthesized as an inhibitor of SHMT1/2 (20), and AGF347 was developed as a multitargeted one-carbon inhibitor at SHMT1/2, with additional inhibition of *de novo* purine biosynthesis at glycinamide ribonucleotide (GAR) formyltransferase and 5-aminoimidazole-4-carboxamide ribonucleotide (AICAR) formyltransferase (21, 22). SHIN1, a very specific tool compound, has been shown to reduce cell proliferation in various tumor types, including lymphoma (20), rhabdomyosarcoma (23), bladder cancer (24), and lung cancer (25, 26) cell lines. AGF347 has shown promising therapeutic activity *in vitro* and *in vivo*, in both pancreatic and ovarian cancer models (21, 22). As SHIN1 and AGF347 are not clinic-ready, we also used pralatrexate (PRTX), the newest clinically approved antifolate inhibitor of DHFR (27), which targets one-carbon metabolism at a different node. We tested the effects of these three inhibitors on a panel of six Ewing sarcoma cell lines encompassing the spectrum of driver mutations associated with Ewing sarcoma development (Supplementary Table S1). Both SHIN1 and AGF347 were effective in inhibiting the proliferation of Ewing sarcoma cells, with EC_50_s in the low micromolar range (Fig. 3A and B). Interestingly, all cell lines responded very similarly to the inhibitors, independent of the mutations accompanying the fusion oncogene Ewing sarcoma::FLI1. PRTX, the most potent antifolate tested, completely suppressed cell growth at low nanomolar concentrations (Fig. 3C; Table 1).

**Table 1:** EC_50_ of the three inhibitors in Ewing sarcoma cell lines

EC_50_	SHIN1 (µmol/L)	AGF347 (µmol/L)	PRTX (nmol/L)
CHLA-10	3.50 ± 0.07	0.23 ± 0.04	0.92 ± 0.05
MHH-ES-1	7.71 ± 0.05	3.22 ± 0.05	0.93 ± 0.04
SK-ES-1	5.47 ± 0.07	1.46 ± 0.04	0.97 ± 0.06
TC-32	1.14 ± 0.05	2.00 ± 0.06	2.75 ± 0.06
TC-71	4.59 ± 0.04	1.53 ± 0.04	0.94 ± 0.04
RD-ES	5.23 ± 0.04	3.41 ± 0.04	0.74 ± 0.07

To assess the effect of long-term pharmacologic inhibition of one-carbon metabolism on Ewing sarcoma cell proliferation, we performed colony-forming assays on a panel of Ewing sarcoma cell lines treated with SHIN1, AGF347, or PRTX at concentrations roughly corresponding to their 72-hour EC_70_. After 2 weeks, colony formation was completely suppressed by these antifolates (Fig. 3D). These data significantly extend the genetic approaches described in Fig. 2 and further suggest that in Ewing sarcoma cells, one-carbon pathway inhibition leads to profound growth suppression.

**Figure 4:**
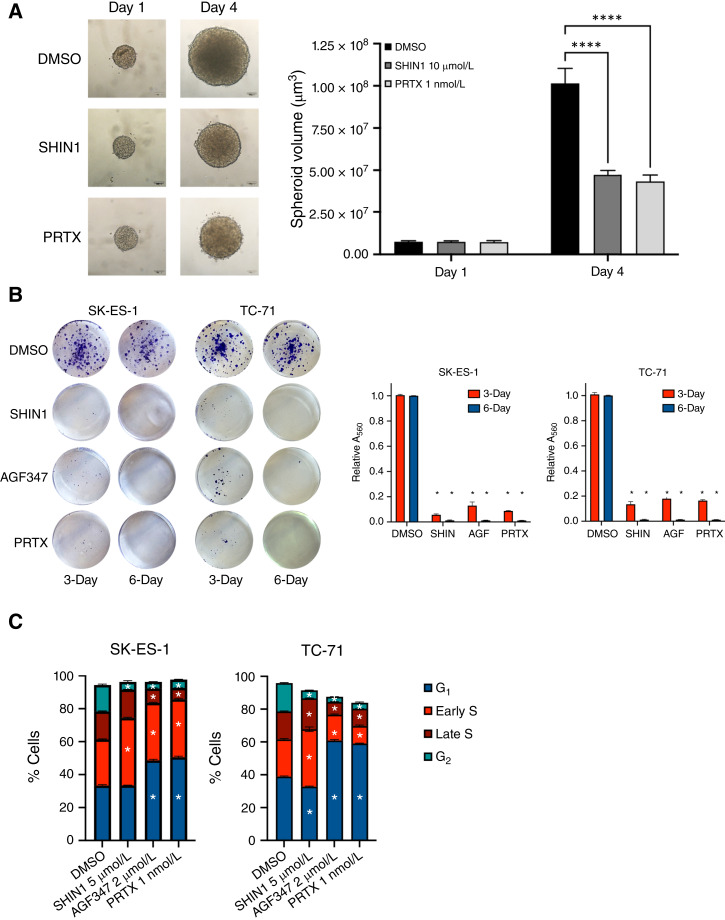
Pharmacologic inhibition of one-carbon metabolism results in inhibition of Ewing sarcoma spheroid growth and colony formation, resulting in G_1_ phase to early S-phase arrest. **A,** Analysis of the inhibitory effect of SHIN1 and PRTX on the growth of SK-ES-1 spheroids. *, *P* < 0.001. **B,** Colony-forming assay of SK-ES-1 and TC-71 cells treated with SHIN1 (10 µmol/L), AGF347 (10 µmol/L), and PRTX (1 nmol/L) for 6 days and then grown without drugs for seven additional days. Quantitative results are shown in the graph to the right. *, *P* < 0.0001. **C,** Cell-cycle analysis of SK-ES-1 and TC-71 cells treated with the indicated compounds for 48 hours. *, *P* < 0.01 from replicate experiments.

Next, we tested the effect of one-carbon metabolism inhibition in SK-ES-1 three-dimensional spheroid models, which recapitulate the biology of solid tumors more closely than classical cell culture in a dish. After 3 days of drug treatment, we found that both SHIN1 and PRTX inhibited spheroid growth by more than 50%, further validating the efficacy of targeting different nodes of one-carbon metabolism in Ewing sarcoma (Fig. 4A).

To determine whether the growth suppression consequent to one-carbon pathway inhibition is reversible upon drug “washout,” we treated SK-ES-1 and TC-71 Ewing sarcoma cells with one-carbon metabolism inhibitors for 3 or 6 days and then removed the compounds for an additional 7 days. Strikingly, both cell lines progressively lost their ability to resume proliferation (>90% reduction after 6-day treatment, Fig. 4B). These results strongly suggest that in Ewing sarcoma cells, one-carbon pathway inhibition leads to a state of growth suppression that is progressively less reversible upon drug removal.

To identify the mechanisms leading to growth suppression upon inhibition of the one-carbon metabolic pathway, we measured the effects of SHIN1, AGF347, or PRTX treatment on the induction of cell death using a lactate dehydrogenase release assay. We did not find any evidence of cell death upon treatment with these inhibitors (Supplementary Fig. S3A). We then analyzed the effects of the three inhibitors on the cell-cycle distribution of SK-ES-1 and TC-71 cells and observed a significant reduction of cells in the G_2_ phase and an accumulation of cells in the G_1_ phase and/or early S-phase of the cell cycle. These data suggest that inhibition of one-carbon metabolism impairs the ability of Ewing sarcoma cells to complete DNA replication (Fig. 4C).

### One-carbon pathway inhibition leads to nucleotide depletion

Mitochondrial one-carbon metabolism from serine results in the synthesis of glycine (via SHMT2), NADH (via MTHFD2), and formate (via MTHFD1L2; refs. 7–9). Formate passes to the cytosol, in which it participates in cellular anabolism. Inhibition of the one-carbon pathway at the SHMT2 node is predicted to lead to two critical metabolic deficiencies. These include depletion of formate, which is the principal source of one-carbon units for *de novo* nucleotide synthesis in the cytosol, and glycine, a critical amino acid for the synthesis of proteins and purine nucleotides (Fig. 1A).

**Figure 5:**
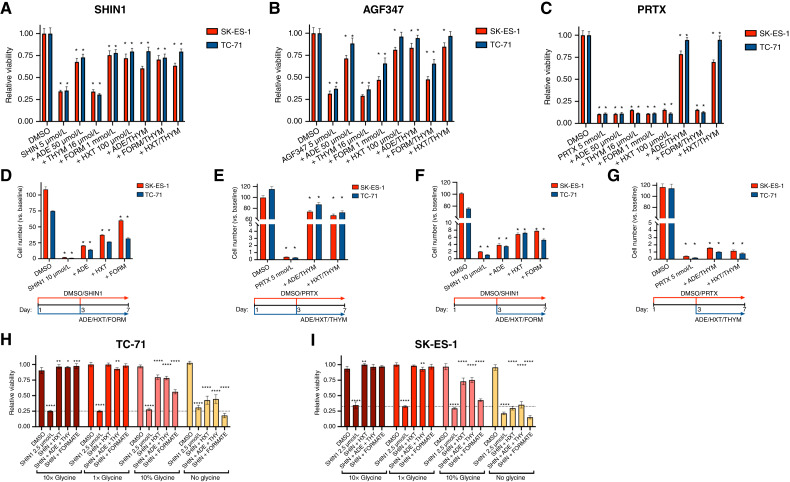
Progressive loss of the ability of relevant metabolites to rescue the proliferation of Ewing sarcoma cells after one-carbon metabolism inhibition. **A–C,** Rescue by exogenous adenine, thymidine, formate, and hypoxanthine addition of the growth inhibition induced by 72 hours of treatment of SK-ES-1 and TC-71 cells with one-carbon inhibitors. **D** and **E,** Rescue by metabolite supplementation of the growth inhibition induced by 7-day treatment of SK-ES-1 and TC-71 cells with SHIN1 (**D**) and PRTX (**E**). **F** and **G,** Rescue effect of delayed (at day 3) metabolite addition on the growth inhibition induced by 7-day treatment of SK-ES-1 and TC-71 cells with SHIN1 (**F**) and PRTX (**G**). **A–G,** *, *P* < 0.001 vs. DMSO. **H** and **I,** Exogenous glycine deprivation impairs the ability of hypoxanthine, adenine, thymidine, and formate (concentrations as in **A–C**) to rescue the proliferation impairment consequent to SHMT1/2 inhibition with SHIN1. Glycine-free RPMI 1640 was supplemented with 1.33 mmol/L (10×), 133 µmol/L (1×), or 13.3 µmol/L (10%) glycine. *, *P* < 0.05; **, *P* < 0.01; ***, *P* < 0.001; ****, *P* < 0.0001.

To further define the relative impact of inhibiting one-carbon metabolism on these cytosolic pathways in Ewing sarcoma cells, we first tested whether providing exogenous metabolites can rescue cells from the growth suppression caused by pharmacologic inhibition of SHMT1 and SHMT2. Supplementation of the growth medium with 1 mmol/L formate, in the presence of glycine (133 µmol/L), partially restored the proliferative ability of Ewing sarcoma cells treated with SHIN1 (∼70%; Fig. 5A) and/or AGF347 (∼60%; Fig. 5B). The additional impact of AGF347 in these experiments likely reflects the direct targeting of *de novo* purine biosynthesis at GAR and AICAR formyltransferases (21), which are downstream of formate.

To distinguish between the drug effects on one-carbon metabolism leading to purine nucleotide versus thymidylate synthesis, we also tested the protective impact of exogenous adenine or hypoxanthine, which provide substrates for the purine salvage pathway; results were compared with those for exogenous thymidine, which is metabolized to thymidylate, thus circumventing the thymidylate synthase reaction. Both adenine and hypoxanthine substantially reversed the proliferative block induced by SHIN1 and AGF347 in Ewing sarcoma cells. This suggests that in the presence of glycine, purine depletion is a major consequence of inhibiting one-carbon metabolism by these agents (Fig. 5A and B). Thymidine supplementation did not rescue the proliferation deficit and did not alter the extent of the rescue induced by hypoxanthine and adenine (Fig. 5A and B). Thus, thymidylate levels do not seem to be limiting for the proliferation of Ewing sarcoma cells treated with SHIN1 or AGF347. In contrast to our findings with SHIN1 or AGF347, rescue from the inhibitory effects of PRTX required a combination of adenine (or hypoxanthine) with thymidine, results entirely consistent with its inhibition of DHFR (Fig. 5C).

To test whether exogenous metabolite supplementation can also prevent the antiproliferative effects of one-carbon metabolism inhibition at SHMT2 or DHFR over a longer period of time, we treated SK-ES-1 and TC-71 cells with a high dose of SHIN1 (10 µmol/L) or PRTX (5 nmol/L), alone or in the presence of rescuing metabolites, for 7 days, with a complete change of medium and compounds after 3 days. Exogenous adenine, hypoxanthine, and formate were only able to partially prevent growth suppression. This strongly suggests that upon prolonged inhibition of one-carbon metabolism at SHMT1/2, purine depletion is accompanied by deficiencies in additional metabolites or biological processes, which become limiting for Ewing sarcoma cell proliferation (Fig. 5D). The effect of prolonged treatment with PRTX could, in contrast, be prevented to a greater extent by supplementation with adenine (or hypoxanthine) and thymidine (Fig. 5E).

Next, we tested whether the growth suppression caused by inhibition of one-carbon metabolism is reversible after being fully established. To this end, we supplemented purine-generating metabolites 3 days after treatment with a high dose of SHIN1 and measured cell proliferation after an additional 4 days of combined treatment. In the presence of adenine, hypoxanthine, or formate, Ewing sarcoma cells resumed a low level of proliferation despite continuous SHIN1 presence, indicating that within the time frame analyzed, purine availability can only partially reverse the preexisting growth arrest induced by inhibition of one-carbon metabolism at the SHMT level (Fig. 5F). Further extending the preinhibition to 6 days before hypoxanthine supplementation resulted in even lower proliferation recovery, whereas a higher resumption of proliferation was observed if SHIN1 was removed from the growth medium (Supplementary Fig. S3B). Inhibition of proliferation with PRTX led to a more profound effect, which was only nominally reversed by metabolite addition after 3 days of pretreatment (Fig. 5G). These data suggest that inhibition of one-carbon metabolism in Ewing sarcoma cells results in progressively irreversible growth suppression.

In a previous study, we found that thyroid cancer cells become fully auxotrophic for glycine upon inhibition of SHMT1/2 with SHIN1. In the absence of exogenous (i.e., culture medium) glycine (133 µmol/L), the growth suppressive effect of SHIN1 was dramatically amplified, leading to cell death (28). We tested the effect of removing extracellular glycine on the response of Ewing sarcoma cells to SHIN1. Surprisingly, despite the complete inhibition of glycine synthesis from serine, the growth inhibitory effects of SHIN1 did not change with the presence or absence of glycine in the culture medium, and SHIN1-treated Ewing sarcoma cells survived in glycine-free medium (Supplementary Fig. S4). Additionally, supplementation of the culture medium with a 10-fold excess of glycine (1.33 mmol/L) did not change the effect of SHIN1 (Fig. 5H and I). These data suggest that contrary to results with thyroid cancer cells (28), the growth-suppressive effects of one-carbon metabolism inhibitors on Ewing sarcoma cells are largely independent of glycine availability. Ewing sarcoma cells might procure sufficient glycine for basic survival through alternative pathways. However, in the absence of exogenous glycine, supplementation with purine-producing metabolites was unable to restore Ewing sarcoma cell proliferation in the presence of SHIN1, indicating that the hypothetical alternative source of glycine may not be sufficient to meet the demand of all the metabolic processes requiring glycine (Fig. 5H and I).

### Metabolomic analysis of one-carbon flux

**Figure 6:**
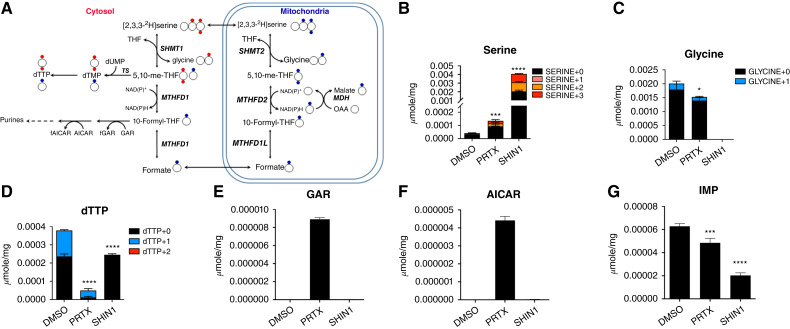
Effects of SHIN1 and PRTX on one-carbon flux through the mitochondrial and cytoplasmic compartments. **A,** Schematic of one-carbon flux through the mitochondrial and cytosol compartments using [2,3,3-^2^H]L-serine. The ^2^H atoms in [2,3,3-^2^H]L-serine (blue circle) in the mitochondria are metabolized to generate [^2^H]formate. [^2^H]Formate in the cytosol is converted to [^2^H]10-formyl THF and [^2^H]5,10-methylene THF, resulting in [^2^H]dTMP and [^2^H]dTTP (as M+1). Reversal of SHMT1 activity in the cytosol results in ^2^H atoms in [2,3,3-^2^H]serine (red) being metabolized to [^2^H]5,10-methylene THF, which is utilized by thymidylate synthase to synthesize [^2^H]dTMP and [^2^H]dTTP (as M+2). **B** and **C,** Total serine (**B**) and glycine (**C**) and the serine-isotopomer distributions in SK-ES-1 cells treated with vehicle (DMSO), 10 nmol/L PRTX, or 10 µmol/L SHIN1. The increase in the M+1 and M+2 serine isotopomers in the presence of the inhibitors is a direct reflection of accumulated [2,3,3-^2^H]L-serine accompanying the loss of SHMT2 activity, combined with low levels of one-carbon flux through SHMT2 and MTHFD2 in mitochondria and SHMT1 in the cytosol. **D,** Total dTTP pools are shown with the isotopomer distributions (M+0, M+1, M+2) for untreated and inhibitor-treated SK-ES-1 cells compared with DMSO-treated cells. **E–G,** GAR (**E**), AICAR (**F**), and inosine monophosphate (IMP; **G**) pools were measured in vehicle and inhibitor-treated cells. An unpaired *t* test was performed for total serine, total glycine, total dTTP, and IMP in comparison with the DMSO-treated sample. *, *P* < 0.05; ***, *P* < 0.001; ****, *P* < 0.0001.

We performed metabolomic analysis of SK-ES-1 cells with [2,3,3-^2^H]L-serine to study the impact of drug treatments on intracellular one-carbon metabolism. SK-ES-1 cells were treated with vehicle (1% DMSO). Cells were treated with PRTX (10 nmol/L) or SHIN1 (10 µmol/L) for 24 hours, followed by [2,3,3-^2^H]L-serine (250 µmol/L) for another 24 hours, and then processed for determinations of total serine and glycine, along with their isotopomer distributions (M+3, M+2, M+1, and M+0 for serine; M+1 and M+0 for glycine; M+n for species with n deuterium atoms). The results for the drug-treated cells were compared with those for vehicle-treated cells. A schematic of [2,3,3-^2^H]L-serine metabolism via SHMT2 and SHMT1 (including distributions of deuterium atoms in downstream metabolites, including dTTP) is included in Fig. 6A.

Treatment of SK-ES-1 cells with PRTX and SHIN1 increased total serine levels 3- and 80-fold, respectively, above the levels in vehicle-treated cells (Fig. 6B). For SHIN1-treated cells, elevated serine pools were accompanied by increased M+3 (180-fold) and M+2 (449-fold) serine isotopomers over vehicle-treated cells. There was a ∼24% decrease in total glycine for cells treated with PRTX. Strikingly, SHIN1 treatment resulted in a complete loss of glycine (Fig. 6C).

The ^2^H-dTTP isotopomer distribution from [2,3,3-^2^H]L-serine was used as a direct measure of relative mitochondrial and cytosolic one-carbon fluxes through SHMT2 and SHMT1 into dTMP (and dTTP; ref. 10). Thus, the hydrogen-1 flux from [2,3,3-^2^H]L-serine to [^2^H]formate via SHMT2 in mitochondria results in M+1 [^2^H]dTTP, whereas the ^2^H flux from [2,3,3-^2^H]L-serine via the reversal (serine-to-glycine) of SHMT1 catalysis in the cytosol results in M+2 [^2^H]dTTP (Fig. 6A; refs. 7, 10, 20, 21). Treatment of SK-ES-1 cells with SHIN1 decreased total dTTP pools (35%), and M+1 dTTP was completely abolished compared with vehicle-treated cells (Fig. 6D). These results support the direct targeting of SHMT2 by SHIN1 (20). Meanwhile, PRTX treatment resulted in a dramatic decrease in both dTTP+0 (95%) and dTTP+1 (73%), most likely due to the potent inhibition of DHFR, resulting in a near-complete loss of THF with downstream effects on one-carbon transfer. Interestingly, there was no detectable M+2 dTTP, suggesting that the SK-ES-1 cells rely primarily on mitochondrial one-carbon metabolism for proliferation.

We also assessed the impact of PRTX and SHIN1 on *de novo* purine biosynthesis in SK-ES-1 cells. We measured accumulated GAR and AICAR (respective substrates for GARFTase and AICARFTase; Fig. 6A) by LC/MS. GAR and AICAR levels were nominal in vehicle- and SHIN1-treated cells but accumulated to significant levels in PRTX-treated cells (Fig. 6E and F). Although no accumulation of GAR and AICAR was observed in either vehicle- or SHIN1-treated SK-ES-1 cells, the basis for this result is likely very different, with normal purine biosynthesis ongoing for the former and a complete shutdown of the purine pathway resulting from glycine depletion for the latter. This is reflected in the levels of inosine monophosphate (Fig. 6I). Inhibition of DHFR by PRTX profoundly decreased purine biosynthesis, reflected in the substantial accumulation of GAR and AICAR intermediates. PRTX and SHIN1 treatments decreased total inosine monophosphate pools by 23% and 68%, respectively (Fig. 6G).

Thus, inhibition of one-carbon metabolism in Ewing sarcoma cells leads to a near-complete cessation of the synthesis of glycine and purines with SHMT1/2 inhibitors and a significant impairment with DHFR inhibition, which has its major impact on dTTP synthesis.

### Effect of SHMT2 depletion on tumor growth *in vivo*

**Figure 7:**
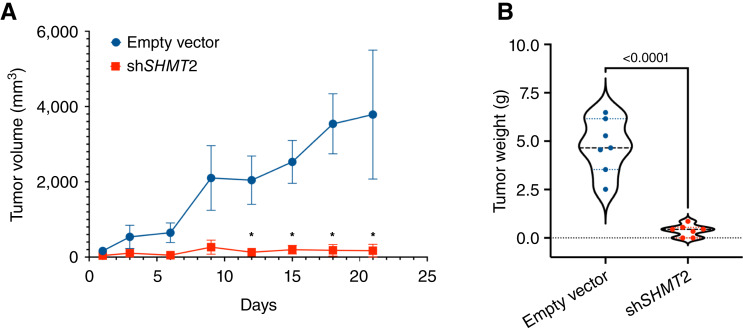
*In vivo* efficacy of genetic one-carbon metabolism inhibition. **A,** shRNA-mediated depletion of *SHMT2* severely impairs *in vivo* tumor growth in SK-ES-1 xenografts, resulting in significantly smaller tumors, as shown by the difference in weight of the excised tumors at the end of the experiment (**B**). * in **A** indicates *P* < 0.01.

To assess *in vivo* the effect of inhibiting mitochondrial one-carbon metabolism in Ewing sarcoma cells, we used SK-ES-1 xenografts expressing either a control shRNA or sh*SHMT2* (doxycycline-inducible) in NOD-*scid IL2r*^*null*^ mice. The two cell lines did not show a difference in their proliferation in the absence of doxycycline (Supplementary Fig. S5). Mice were injected subcutaneously on one flank with the control cells and on the other flank with the SHMT2-depleted cells. The SHMT-depleted cell lines showed a dramatic reduction in tumor growth rate (Fig. 7A) as well as in tumor weight at the end of the experiment (4.74 g for vector-transduced cells vs. 0.38 g for ish*SHMT2*-expressing cells, Fig. 7B), thus providing direct evidence that inhibition of one-carbon metabolism could be a viable treatment strategy for patients with Ewing sarcoma.

## Discussion

Cancer cells undergo various metabolic alterations to adapt to their heightened proliferative demands and their unique microenvironment. These changes enable tumors to sustain rapid growth, survive under conditions of limited oxygen or nutrient supply, and resist stress from their surroundings. These metabolic adaptations frequently create vulnerabilities that are unique to cancer cells, distinguishing tumors from normal cells. As such, they represent valuable targetable liabilities that can be exploited in the development of novel therapeutic strategies (29).

One of the metabolic pathways most often upregulated by tumor cells is one-carbon metabolism, which utilizes serine and dietary folates to generate glycine and THF-bound one-carbon units required for *de novo* nucleotide biosynthesis, NAD(P)H and glutathione production, methionine regeneration, DNA methylation, and translation of mitochondrial proteins (7–9).

*SHMT2* and *MTHFD2*, encoding two key enzymes of the mitochondrial arm of the one-carbon pathway, have been reported to be among the most consistently upregulated metabolic genes in cancer (11, 12). Not surprisingly, this has led to renewed interest in targeting mitochondrial one-carbon metabolism for cancer (20, 25, 30, 31).

Selective inhibitors of these enzymes, however, are still at an early stage of preclinical development, whereas the value of repurposed inhibitors with reported activity against SHMT2 [e.g., sertraline (32), metformin (33)] is difficult to assess due to a lack of target specificity. PRTX, a clinically approved antifolate drug targeting DHFR (27), offers, however, the possibility to test the efficacy of targeting one-carbon metabolism, although at a different node.

In this study, our data shed important new light on the role of one-carbon metabolism in driving the proliferation of Ewing sarcoma cells and demonstrate a remarkable dependence on this pathway, a notion supported by the dramatic effect of genetic and pharmacologic inhibition of one-carbon metabolism. Inhibition of SHMT1/2 or DHFR led to strong cytostatic effects that persisted for several days even when the inhibitor was removed. Whether these cells undergo irreversible senescence or enter a prolonged but still partially reversible state of quiescence remains to be addressed in future work.

When glycine is present, metabolites such as hypoxanthine and adenine, but not thymidine, rescue the proliferation block associated with inhibition of one-carbon metabolism at the level of SHMT1/2. Although in agreement with other studies in which purine synthesis is the specific essential conduit of SHMT1/2 inhibition (20), these data raise the question of why thymidylate does not become limiting, at least in the time frame analyzed, as it does when one-carbon metabolism is inhibited at the level of DHFR with PRTX.

In previous studies, we have shown that inhibition of one-carbon metabolism at SHMT1/2 in anaplastic thyroid cancer cells leads to glycine auxotrophy and rapid cell death when cells are deprived of extracellular glycine (28). Interestingly, this does not seem to be the case for Ewing sarcoma cells as the presence or absence of glycine in the medium does not alter the efficacy of both SHIN1 and AGF347. Although it is possible that alternative intracellular sources of glycine (34) are able to maintain the viability of Ewing sarcoma cells when both one-carbon metabolism–mediated synthesis and extracellular uptake are impaired, the inability of these cells to remain proliferative when adenine or hypoxanthine is supplemented suggests that this alternative source of glycine is still limiting, a notion that offers an interesting targetable liability for future therapeutic applications.

We used a genetic approach to inhibit one-carbon metabolism *in vivo* in an immunocompromised xenograft model. Consistent with our *in vitro* findings, our *in vivo* results strongly suggest that SHMT inhibition is a viable and promising strategy on which to build novel therapeutic approaches to Ewing sarcoma. Although inhibition of one-carbon metabolism in Ewing sarcoma cells seems to be cytostatic, the prolonged growth arrest observed in cell culture suggests the possibility of extended tumor control *in vivo*, a therapeutic goal of utmost importance in advanced, metastatic, or recurrent Ewing sarcoma management. In addition, we believe that these approaches represent a solid foundation for the development of rationally designed synthetic lethal strategies harnessing the liabilities exposed by one-carbon metabolism impairment.

## Supplementary Material

Supplementary Table 1 Supplementary Table 1

Supplementary Figure 1 Overall survival of EWS patients (n=44) expressing high or low levels of MTHFD1L, SHMT1, MTHFD1. Analysis was performed on dataset GSE17679 using R2: Genomics Analysis and Visualization Platform (http://r2.amc.nl).

Supplementary Figure 2 Incucyte live analysis of TC-71 cell proliferation upon depletion of SHMT1 and SHMT2.

Supplementary Figure 3 A) Effect of the indicated treatments on the induction of cell death as measured by LDH release. B) Effect (at day 10) of delayed hypoxanthine addition or SHIN1 removal, both at day 6, on the growth inhibition induced in SK-ES-1 and TC-71 cells SHIN1 treatment.

Supplementary Figure 4 Exogenous glycine deprivation does not alter the efficacy of 72h treatment of EWS cells with SHIN1. Cells were grown in glycine-free RPMI1640 with or without supplementation with 133µM glycine.

Supplementary Figure 5 Incucyte live analysis of the proliferation in the absence of doxycycline of the SK-ES-1 lines utilized in the in vivo experiment.
