# Supplementary material for: One-Carbon Metabolism Inhibition Depletes Purines and Results in Profound and Prolonged Ewing Sarcoma Growth Suppression
Source: Cancer Res Commun. 2025 Aug 8;5(8):1298–309. doi: 10.1158/2767-9764.CRC-25-0218 (PMC12332480; doi:10.1158/2767-9764.CRC-25-0218)
Supplement: Supplementary Table 1 [file crc-25-0218_supplementary_table_1_suppst1.docx]

**Supplementary Table 1**

**Driver mutations in the EWS cell lines used in this study**

|  | FUSION | TP53 | STAG2 | CDKN2A |
| --- | --- | --- | --- | --- |
| CHLA-10 (RRID:CVCL_6583) | EWS::FLI1 | mut | wt | wt |
| MHH-ES-1 (RRID:CVCL_1411) | EWS::FLI1 | mut | mut | wt |
| SK-ES-1 (RRID:CVCL_0627) | EWS::FLI1 | mut | mut | wt |
| TC-32 (RRID:CVCL_7151) | EWS::FLI1 | wt | mut | mut |
| TC-71 (RRID:CVCL_2213) | EWS::FLI1 | mut | wt | mut |
| RD-ES (RRID:CVCL_2169) | EWS::FLI1 | mut | wt | wt |
