# Supplementary material for: One-Carbon Metabolism Inhibition Depletes Purines and Results in Profound and Prolonged Ewing Sarcoma Growth Suppression
Source: Cancer Res Commun. 2025 Aug 8;5(8):1298–309. doi: 10.1158/2767-9764.CRC-25-0218 (PMC12332480; doi:10.1158/2767-9764.CRC-25-0218)
Supplement: Supplementary Figure 1 — Overall survival of EWS patients (n=44) expressing high or low levels of MTHFD1L, SHMT1, MTHFD1. Analysis was performed on dataset GSE17679 using R2: Genomics Analysis and Visualization Platform (http://r2.amc.nl). [file crc-25-0218_supplementary_figure_1_suppsf1.pdf]

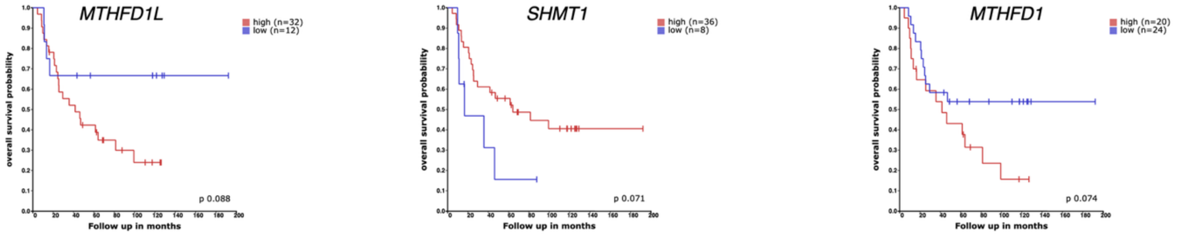

Supplementary Figure 1

Overall survival of EWS patients (n=44) expressing high or low levels of *MTHFD1L*, *SHMT1*, *MTHFD1.* Analysis was performed on dataset GSE17679 using R2: Genomics Analysis and Visualization Platform (http://r2.amc.nl).
