# Supplementary material for: One-Carbon Metabolism Inhibition Depletes Purines and Results in Profound and Prolonged Ewing Sarcoma Growth Suppression
Source: Cancer Res Commun. 2025 Aug 8;5(8):1298–309. doi: 10.1158/2767-9764.CRC-25-0218 (PMC12332480; doi:10.1158/2767-9764.CRC-25-0218)
Supplement: Supplementary Figure 2 — Incucyte live analysis of TC-71 cell proliferation upon depletion of SHMT1 and SHMT2. [file crc-25-0218_supplementary_figure_2_suppsf2.pdf]

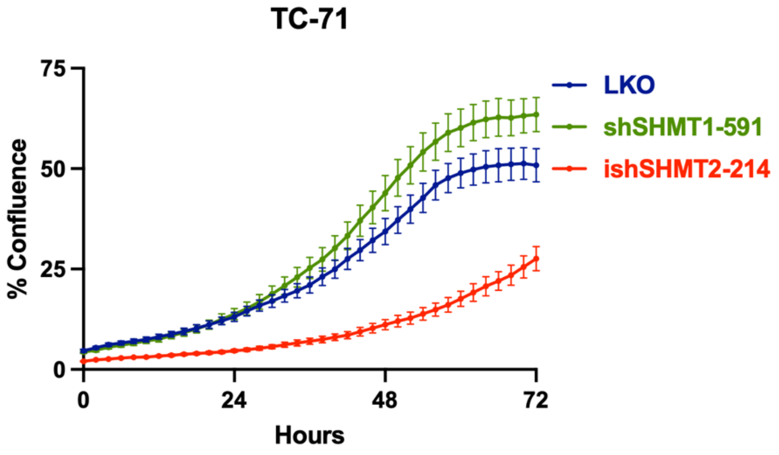

Supplementary Figure 2

Incucyte live analysis of TC-71 cell proliferation upon depletion of *SHMT1* and *SHMT2.*
