# Supplementary material for: One-Carbon Metabolism Inhibition Depletes Purines and Results in Profound and Prolonged Ewing Sarcoma Growth Suppression
Source: Cancer Res Commun. 2025 Aug 8;5(8):1298–309. doi: 10.1158/2767-9764.CRC-25-0218 (PMC12332480; doi:10.1158/2767-9764.CRC-25-0218)
Supplement: Supplementary Figure 3 — A) Effect of the indicated treatments on the induction of cell death as measured by LDH release. B) Effect (at day 10) of delayed hypoxanthine addition or SHIN1 removal, both at day 6, on the growth inhibition induced in SK-ES-1 and TC-71 cells SHIN1 treatment. [file crc-25-0218_supplementary_figure_3_suppsf3.pdf]

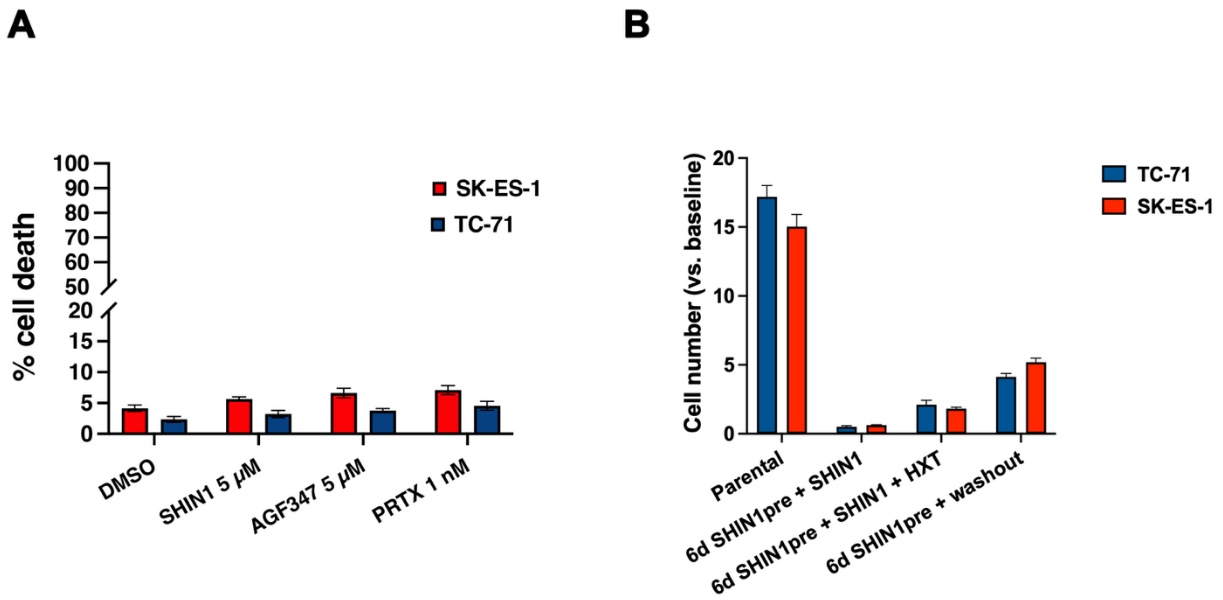

Supplementary Figure 3

A) Effect of the indicated treatments on the induction of cell death as measured by LDH release.

B) Effect (at day 10) of delayed hypoxanthine addition or SHIN1 removal, both at day 6, on the growth inhibition induced in SK-ES-1 and TC-71 cells SHIN1 treatment.
