# Supplementary material for: One-Carbon Metabolism Inhibition Depletes Purines and Results in Profound and Prolonged Ewing Sarcoma Growth Suppression
Source: Cancer Res Commun. 2025 Aug 8;5(8):1298–309. doi: 10.1158/2767-9764.CRC-25-0218 (PMC12332480; doi:10.1158/2767-9764.CRC-25-0218)
Supplement: Supplementary Figure 4 — Exogenous glycine deprivation does not alter the efficacy of 72h treatment of EWS cells with SHIN1. Cells were grown in glycine-free RPMI1640 with or without supplementation with 133µM glycine. [file crc-25-0218_supplementary_figure_4_suppsf4.pdf]

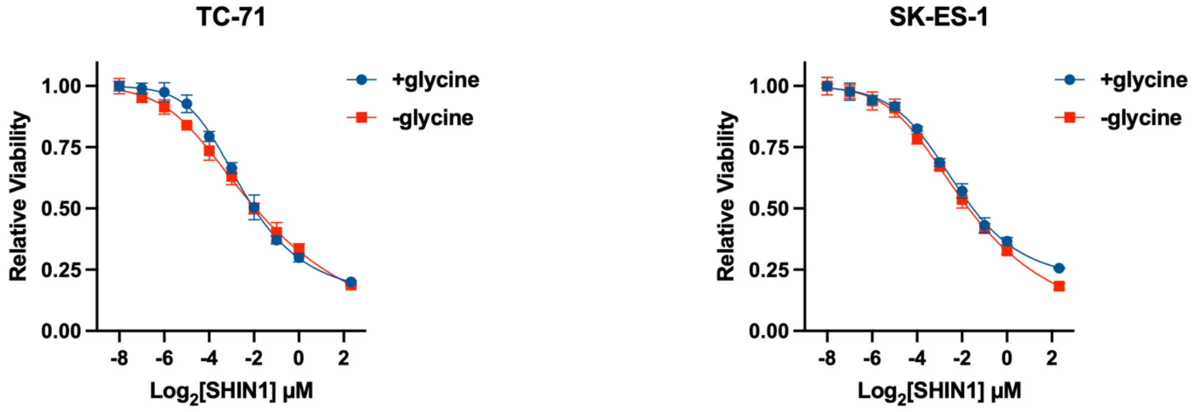

Supplementary Figure 4
Exogenous glycine deprivation does not alter the efficacy of 72h treatment of EWS cells with SHIN1. Cells were grown in glycine-free RPMI1640 with or without supplementation with 133μM glycine.
