# Supplementary material for: One-Carbon Metabolism Inhibition Depletes Purines and Results in Profound and Prolonged Ewing Sarcoma Growth Suppression
Source: Cancer Res Commun. 2025 Aug 8;5(8):1298–309. doi: 10.1158/2767-9764.CRC-25-0218 (PMC12332480; doi:10.1158/2767-9764.CRC-25-0218)
Supplement: Supplementary Figure 5 — Incucyte live analysis of the proliferation in the absence of doxycycline of the SK-ES-1 lines utilized in the in vivo experiment. [file crc-25-0218_supplementary_figure_5_suppsf5.pdf]

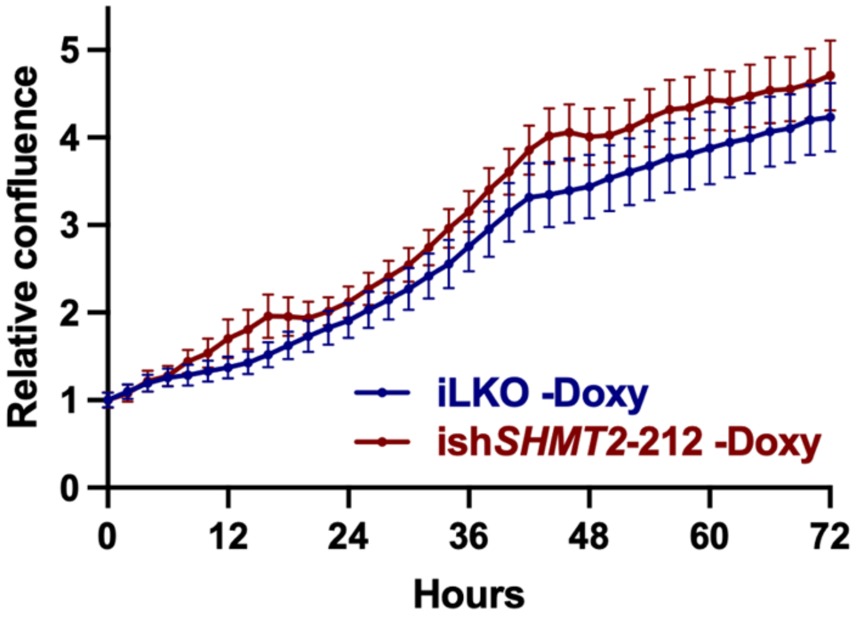

Supplementary Figure 5
Incucyte live analysis of the proliferation in the absence of doxycycline of the SK-ES-1 lines utilized in the in vivo experiment.
